# Relationship between doses of antihypertensive drugs and left ventricular mass index changes in hemodialysis patients in a Japanese cohort

**DOI:** 10.1080/0886022X.2021.1872626

**Published:** 2021-01-17

**Authors:** Fumiya Kitamura, Makoto Yamaguchi, Takayuki Katsuno, Hironobu Nobata, Shiho Iwagaitsu, Hirokazu Sugiyama, Hiroshi Kinashi, Shogo Banno, Masahiko Ando, Yoko Kubo, Yasumasa Kawade, Iwashima Shigejiro, Yutaka Ito, Takuji Ishimoto, Yasuhiko Ito

**Affiliations:** a Department of Nephrology and Rheumatology, Aichi Medical University, Nagakute, Japan; b Data Coordinating Center, Department of Advanced Medicine, Nagoya University Hospital, Nagoya, Japan; c Department of Preventive Medicine, Nagoya University Graduate School of Medicine, Nagoya, Japan; d Suzuka Kidney Clinic, Josuikai Group, Suzuka, Japan; e Yokkaichi Kidney Clinic, Josuikai Group, Yokkaichi, Japan; f Department of Nephrology and Renal Replacement Therapy, Nagoya University Graduate School of Medicine, Nagoya, Japan

**Keywords:** Antihypertensive, left ventricular mass index, hemodialysis, cardiovascular disease

## Abstract

Left ventricular hypertrophy commonly occurs in dialysis patients and is associated with a risk of developing cardiovascular disease events and all-cause mortality. Although hypertension treatment reduces left ventricular mass index (LVMI) in hemodialysis patients, the relationships of prescription pattern, dose, and changes in the dose of antihypertensive drugs with LVMI have not been completely elucidated. Here, we hypothesized that volume reduction would lead to a decrease in the antihypertensive drug dose and subsequently to a reduction in LVMI; conversely, fluid retention would lead to an increase in the antihypertensive drug use and, subsequently, to LVMI progression. To assess this hypothesis, we investigated the relationship between changes in the dose of antihypertensive drugs and subsequent changes in LVMI in 240 patients who had just started hemodialysis using a retrospective hemodialysis cohort in Japan. Using multiple linear regression analysis, we assessed the association between changes in the antihypertensive drug dose over 1 year after hemodialysis initiation and changes in LVMI during this period. A decrease and an increase in the antihypertensive drug dose were significantly associated with a reduction in LVMI (vs. no change; β  = – 17.386, *p* < .001) and LVMI progression (vs. no change; β  = 16.192, *p* < .001), respectively. In conclusion, our findings suggested that volume reduction, leading to a decrease in the use of antihypertensive drugs, is a therapeutic strategy in patients undergoing hemodialysis to prevent LVMI progression.

## Introduction

Cardiovascular diseases (CVDs) are the most frequent causes of death among hemodialysis (HD) patients [1,2]. Left ventricular hypertrophy (LVH) commonly occurs in dialysis patients and is associated with the risk of developing CVD events and all-cause mortality [3,4]. The mechanisms underlying the development and progression of LVH are multifactorial and include hypertension, volume overload, obesity, arterial stiffening, anemia, and metabolic and humoral abnormalities [5]. Previous studies have shown that hypertension treatment reduces LVH, leading to good prognosis in the general population [6] and HD patients [7–10]. The treatment strategy for hypertension in HD patients includes achieving a euvolemic state and using antihypertensive medications. Targeting dry weight with euvolemia may facilitate high blood pressure (BP) control, leading to a reduction in the use of antihypertensive drugs [11–14]. However, several antihypertensive medications (ranging from 2.8 to 4.0) are often prescribed to HD patients to lower BP [15]. One reason underlying this decision is the lack of a definite index for controlling the volume status, leading to difficulty in achieving euvolemia and, consequently, a reduction in the use of antihypertensive drugs. Furthermore, it is unclear whether extracellular fluid overload can cause LVH progression when BP is controlled by antihypertensive drugs. To date, no previous studies have focused on the relationship between the prescription pattern of antihypertensive drugs, dose, and changes in the antihypertensive medication dose and left ventricular (LV) structure.

In the present study, we hypothesized that achieving euvolemia would lead to a decrease in the dose of antihypertensive drugs and, subsequently, to a reduction in LV mass index (LVMI). Conversely, fluid retention would lead to an increase in the use of antihypertensive drugs and, subsequently, to LVMI progression. To assess this hypothesis, we first investigated the association between the dose of antihypertensive drugs at the time of HD initiation and LVMI. Then, we examined the changes in the dose of antihypertensive drugs and subsequent LVMI changes using a retrospective longitudinal cohort that included hypertensive patients who had just started HD in Japan.

## Materials and methods

### Participants

**Figure 1.**
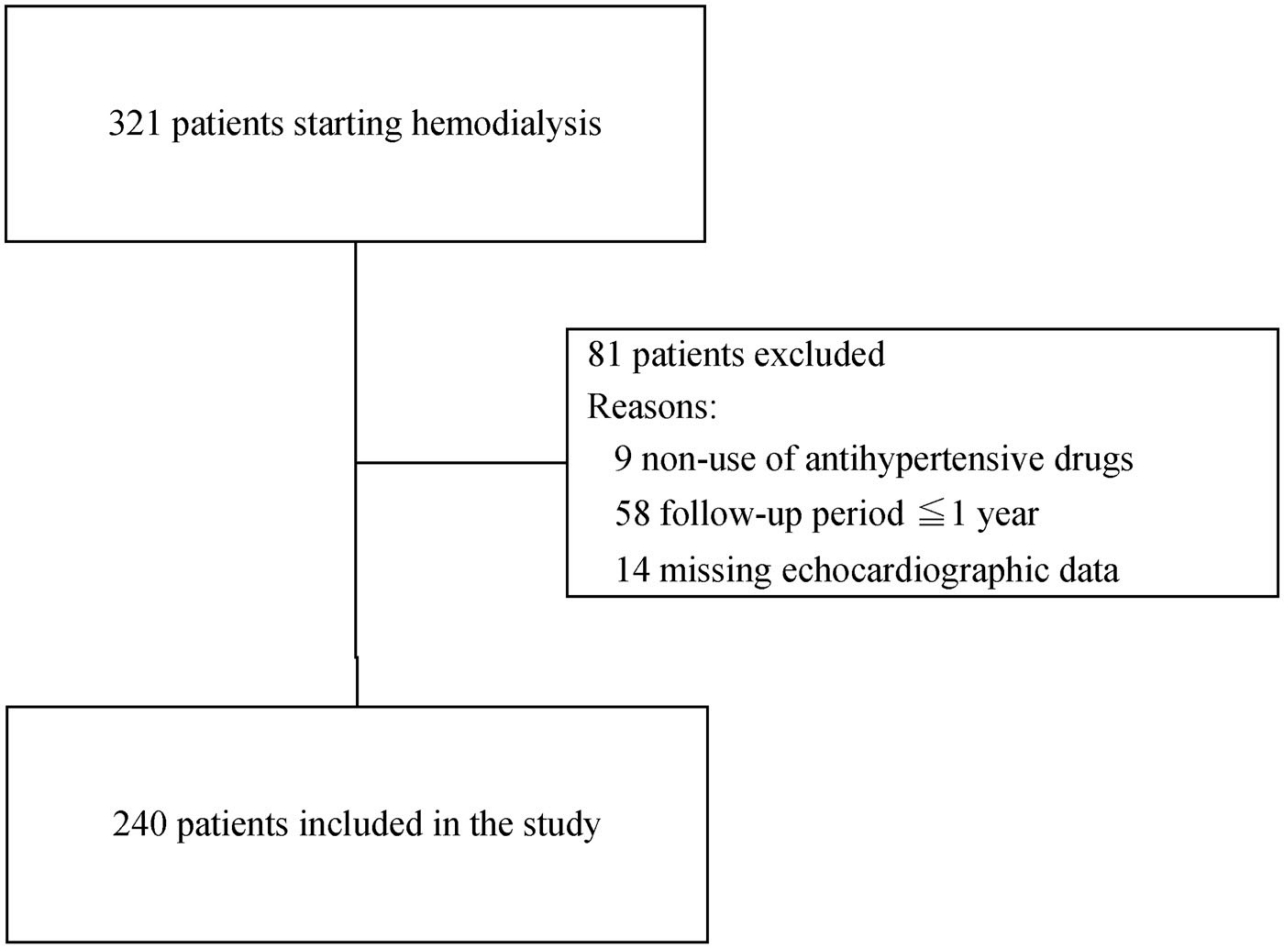
Flow diagram of patient selection.

This cohort study included adult hypertensive HD patients who had received at least one antihypertensive drug upon starting HD and had undergone echocardiography at least twice during an observation period of approximately 1 year. Among 321 patients who had newly started HD (from 2009 to 2014) in two HD centers of the Josuikai group in Japan, we excluded those who had not taken antihypertensive drugs (*n* = 9) and those who could not be reexamined with another echocardiography (*n* = 58). The first echocardiography was performed within 3 months after HD initiation, whereas the second echocardiography was carried out after 1 year. Finally, after excluding 14 patients with missing echocardiographic data, a total of 240 patients were included in the present study (Figure 1). All data were fully anonymized and the ethics committee of the Josuikai group (approval number: 19-1) waived the requirement for patient informed consent owing to the retrospective nature of this study. The anonymous data set are shown in Supplementary Table 1.

**Table 1.** Baseline characteristics at the time of the first cardiac echocardiography (*n* = 240).

Variables	Overall (*n* = 240)	DD*D* ≤ 1 (*n* = 20)	1 < DD*D* ≤ 3 (*n* = 159)	DD*D* > 3 (*n* = 61)	*p*-value
Age (year)	66 (58–74)	64 (56–74)	66 (57–73)	66 (61–75)	.588
Male (N (%))	158 (65.8)	11 (55.0)	105 (66.0)	42 (68.9)	.524
BMI (kg/m^2^)	21.8 (19.6–24.4)	21.8 (19.5–26.6)	21.1 (19.3–23.5)	22.4 (21.1–25.4)	.012
Comorbidities					
Diabetes mellitus	127 (52.9)	11 (55.0)	87 (54.7)	29 (47.5)	.622
Previous cardiovascular diseases	41 (17.1)	3 (15.0)	24 (15.1)	14 (23.0)	.370
Cause of kidney disease					.637
Diabetic nephropathy	122 (50.8)	11 (55.0)	82 (51.6)	29 (47.5)	
Glomerulonephritis	34 (14.2)	2 (10.0)	25 (15.7)	7 (11.5)	
Hypertensive nephrosclerosis	21 (8.8)	0 (0.0)	13 (8.2)	8 (13.1)	
Others	63 (26.3)	7 (35.0)	39 (24.5)	17 (27.9)	
Vascular access type					.336
AVF	214 (89.2)	16 (80.0)	142 (89.3)	56 (91.8)	
AVG	26 (10.8)	4 (20.0)	17 (10.7)	5 (8.2)	
HD duration (h/week)	12.0 (12.0–15.0)	13.5 (12.0–15.0)	12.0 (12.0–15.0)	12.0 (12.0–13.5)	.492
Single-pool Kt/V	1.60 (1.41–1.79)	1.76 (1.49–1.839)	1.56 (1.394–1.79)	1.70 (1.45–1.80)	.148
Pre-dialysis systolic blood pressure (mmHg)	157 (142–170)	156 (141–170)	154 (141–169)	160 (147–173)	.583
Post-dialysis systolic blood pressure (mmHg)	138 (121–148)	137 (120–143)	138 (123–148)	139 (119–153)	.702
Hemoglobin (g/dL)	10.9 (10.5–11.4)	11.1 (10.9–11.3)	10.9 (10.5–11.4)	10.9 (10.4–11.5)	.813
Serum albumin (g/L)	3.7 (3.4–3.9)	3.8 (3.5–4.1)	3.7 (3.4–3.9)	3.7 (3.4–3.8)	.450
Serum potassium (mEq/L)	4.4 (3.9–4.9)	4.5 (4.1–4.9)	4.4 (3.9–4.9)	4.3 (3.6–4.8)	.263
Serum creatinine (mg/dL)	9.3 (7.8–10.9)	9.0 (8.3–10.5)	9.6 (8.0–11.3)	8.7 (7.3–10.7)	.087
Blood urea nitrogen (mg/dL)	55 (49–64)	56 (49–63)	56 (49–63)	57 (49–65)	.945
Serum magnesium (mg/dL)	2.5 (2.3–2.6)	2.4 (2.2–2.6)	2.5 (2.3–2.6)	2.5 (2.3–2.7)	.334
Serum calcium (mg/dL)	8.9 (8.6–9.3)	8.8 (8.5–9.1)	8.9 (8.5–9.3)	9.0 (8.7–9.4)	.247
Serum phosphate (mg/dL)	4.7 (4.1–5.4)	5.0 (4.2–6.0)	4.6 (4.1–5.3)	4.7 (4.2–5.5)	.290
Serum intact PTH (pg/mL)	105 (70–170)	107 (72–148)	108 (66–177)	103 (76–160)	.727
CRP (mg/dL)	.09 (.05–.25)	.08 (.05–.14)	.07 (.05–.24)	.12 (.06–.35)	.122
LDL-cholesterol (mg/dL)	81 (66–99)	78 (68–91)	80 (62–101)	87 (70–100)	.393
Antihypertensive drugs					
Dose of antihypertensive drugs	3 (2–3.5)	1 (1–1)	2.5 (2–3)	4 (3.8–4)	<.001
RAS blocker	217 (90.4)	17 (85.0)	143 (89.9)	57 (93.4)	.506
Calcium channel blocker	226 (94.2)	16 (80.0)	152 (95.6)	58 (95.1)	.018
β-blocker	25 (10.4)	4 (20.0)	5 (3.1)	16 (26.2)	<.001
α-blocker	13 (5.4)	0 (0.0)	4 (2.5)	9 (14.8)	<.001
Other drugs					
Vitamin D	213 (88.8)	17 (85.0)	145 (91.2)	51 (83.6)	.241
Phosphate binder	225 (93.8)	18 (90.0)	148 (93.1)	59 (96.7)	.468
HMG-CoA reductase inhibitor	52 (21.7)	3 (15.0)	28 (17.6)	21 (34.4)	.014
Calcimimetics	38 (15.8)	3 (15.0)	27 (17.0)	8 (13.1)	.777
Dose of erythropoietin (U/week)	3750 (2250–6000)	3375 (1688–6000)	3000 (2250–6000)	3750 (2250–7500)	.666
Cardiothoracic ratio (%)	49 (47–54)	45 (42–48)	48 (46–53)	51 (48–55)	<.001

AVF: arteriovenous fistula; AVG: arteriovenous graft; BMI: body mass index; CRP: C-reactive protein; DDD: defined daily dose; PTH: parathyroid hormone; RAS: renin-angiotensin system. Medians (interquartile ranges), categorical values are expressed as numbers (proportions).

### Measurements

Clinical data were obtained from medical records. The following baseline characteristics were defined at the time of the first echocardiography: age, sex, the primary cause of end-stage renal disease, comorbidities, diabetes, history of previous CVD including coronary heart disease (angina and myocardial infarction), arrhythmia including atrial fibrillation, cardiac arrest, congestive heart failure, and valvular heart disease. We also considered other cardiovascular conditions, such as both pre- and post-dialysis BP, body weight, vascular access type (including arteriovenous fistula or prosthetic graft), weekly erythropoiesis-stimulating agents, dosage, single-pool Kt/V, duration of dialysis treatment (hours per week), and serum laboratory data (including hemoglobin, serum albumin, C-reactive protein, serum creatinine, blood urea nitrogen, serum calcium, serum phosphate, intact parathyroid hormone, and serum magnesium levels). Chest radiographs were used to examine pre-dialysis after a 2-day interdialytic interval in an upright posterior-anterior view, according to the Japanese guidelines [16]. Cardiothoracic ratio, the maximal horizontal diameter of the heart divided by the horizontal inner width of the rib cage, was measured.

### Antihypertensive drugs during the observation period

Prescriptions of antihypertensive medications were obtained from medical records including the renin-angiotensin system (RAS) blockers (angiotensin-converting enzyme inhibitors and angiotensin receptor blockers), β-blockers (selective, nonselective, and α- and β-blocker agents), calcium channel blockers (dihydropyridines and non-dihydropyridines), and centrally acting antiadrenergic agents. Each daily dose was converted to a standardized daily dose based on the corresponding defined daily dose (DDD) both at baseline and 1 year, as proposed by previous studies [17,18].

For example, when a person is receiving valsartan 80 mg (DDD = 80 mg) and amlodipine 10 mg (DDD = 5 mg) per day, he/she is reaching a total score of 3 DDDs.

### Echocardiography

Transthoracic echocardiography was performed according to the American Society of Echocardiography guidelines at baseline and after 1 year [19]. The first echocardiography was performed within 3 months after HD initiation (median of 1.2 [.8–1.4] months) in all patients.

LV mass was calculated using a formula recommended by the American Society of Echocardiography [19] and indexed according to the body surface area. LVH was defined as LVMI by a body surface area of ≥115 and ≥95 g/m^2^ in men and women, respectively [20]. LV outflow velocity was obtained from the apical position. Early and atrial mitral inflow velocities were obtained with a signal positioned at the tip of the mitral leaflets. LV ejection fraction was calculated by the modified biplane (Simpson method) [21].

### Exposure and outcomes

The primary exposure of interest was the change in DDD drugs over 1 year after starting HD. The primary outcome of interest was the change in LVMI (per g/m^2^) over 1 year.

### Statistical analyses

Patients were stratified into three DDD categories at baseline, namely ≤1, 1–3, and >3, as previously reported [22]. The baseline characteristics, echocardiography results, changes in LVMI, cardiothoracic ratio, and pre-dialysis BP over 1 year were compared among these three groups using the Kruskal–Wallis test followed by Mann–Whitney post-hoc analysis with Bonferroni correction or Pearson's chi-square test. To determine the factors that were independently associated with the outcome, we examined the change in LVMI over a period of 1 year using univariate linear regression analysis. Multiple linear regression analysis for the LVMI changes was performed with adjustment for all clinical factors that were included in the univariate model.

The participants were divided into the following three categories according to the DDD changes over 1 year after HD initiation: decrease, no change, or increase. In each group, the LVMI changes were assessed using the paired *t*-test.

The level of statistical significance was set at *p* < .05. All statistical analyses were performed using JMP version 14.0.0 (SAS Institute, Cary, NC, USA), SAS version 9.4 (SAS Institute, Inc, Cary, NC), and STATA version 13.0 (StataCorp LP, College Station, TX, USA).

## Results

### Baseline characteristics and LV measurements of study participants

The baseline characteristics of the study participants and their LV measurements were stratified according to the three DDD categories summarized in Tables 1 and 2, respectively. The present study included 20 (8.3%; DDD ≤ 1), 159 (66.3%; 1 < DDD ≤ 3), and 61 patients (25.4%; DDD > 3). Patients with higher DDD had also higher body mass index (*p* = .032), level of the LVMI/body surface area (*p* < .001), the prevalence of LVH (*p* < .001), cardiothoracic ratio (*p* < .001), and prevalence of HMG-CoA reductase inhibitor use (*p* = .014) compared to those with lower DDD. The other findings were comparable among the groups.

**Table 2.** LV measurements and indices at baseline.

Variables	Overall (*n* = 240)	DD*D* ≤ 1 (*n* = 20)	1 < DD*D* ≤ 3 (*n* = 159)	DD*D* > 3 (*n* = 61)	*p-*value
LV measurements and indices					
LVMI (g/m^2^)	119 (99–145)	75 (67–88)	116 (99–135)	146 (119–172)	<.001
LVH (%)	151 (62.9)	0 (0.0)	95 (59.8)	56 (91.8)	<.001
LVEF (%)	69 (63–75)	71 (63–78)	69 (64–75)	69 (62–74)	.263
AOD (mm)	31 (27–34)	30 (26–32)	30 (27–34)	32 (29–35)	.098
LAD (mm)	36 (32–39)	31 (25–36)	35 (31–39)	37 (35–42)	<.001
LVDd (mm)	42 (37–47)	34 (32–38)	43 (37–47)	44 (40–52)	<.001
LVDs (mm)	26 (22–30)	21 (20–22)	26 (22–30)	27 (25–30)	<.001
IVS (mm)	12 (11–14)	11 (9–13)	12 (11–14)	13 (12–15)	<.001
LVPW (mm)	12 (11–13)	11 (10–13)	12 (11–13)	13 (12–14)	<.001
Mitral inflow E (cm/s)	65 (53–81)	74 (58–83)	66 (51–83)	63 (53–74)	.292
Mitral inflow A (cm/s)	88 (78–104)	90 (78–96)	87 (77–105)	91 (78–100)	.795
E/A	.72 (.60–.86)	0.95 (.70–1.11)	.72 (.59–.85)	.66 (.57–.76)	.018
E/e′	12.3 (9.1–14.7)	9.9 (8.4–17.2)	12.5 (9.2–13.8)	12.2 (8.8–15.7)	.932
Left atrial volume index (mL/m^2^)	16.8 (13.5–21.5)	15.5 (12.5–18.7)	17.2 (13.3–22.3)	16.6 (14.1–20.0)	.689

AOD: aortic end-diastolic diameter; E/A: the ratio of left ventricular early-diastolic inflow velocity (E) to atrial systolic velocity (A); e′: early-diastolic mitral annular velocity; E/e′: the ratio of E to e′; EF: ejection fraction; IVS: interventricular septum; LAD: left atrial dimension; LV: left ventricular; LVDd: left ventricular internal dimension in diastole; LVDs: left ventricular internal dimension in systole; LVH: LV hypertrophy; LVMI: LV mass index; LVPW: left ventricular posterior wall thickness. Medians (interquartile ranges), categorical values are expressed as numbers (proportions).

### Antihypertensive drugs

At baseline, 217 (90.4%) and 226 (94.2%) participants had RAS and calcium channel blockers, respectively. The proportion of RAS and calcium channel blockers was comparable between the groups. Over a period of 1 year after HD initiation, in the DDD ≤ 1 group, eight (40%) patients demonstrated an increase in DDD. In the DDD > 3 group, 40 (80.3%) patients had a decrease in DDD. In the 1 < DDD ≤ 3 group, 105 (66%) patients exhibited no change in DDD (Table 3).

**Table 3.** Change in DDD over a period of 1 year.

Variables	DD*D* ≤ 1 (*n* = 20)	1 < DD*D* ≤ 3 (*n* = 159)	DD*D* > 3 (*n* = 61)	*p*-value
Dose of antihypertensive drugs at baseline	1 (1–1)	2.5 (2–3)	4 (3.8–4)	<.001
Dose of antihypertensive drugs at 1 year	1 (.6–2)	2.5 (2–3)	3 (2.5–3)	<.001
Change in DDD over a period of 1 year				<.001
Decrease	5 (25.0)	29 (18.2)	49 (80.3)	
No change	7 (35.0)	105 (66.0)	7 (11.5)	
Increase	8 (40.0)	25 (15.7)	5 (8.2)	

DDD: defined daily dose. Medians (interquartile ranges), categorical values are expressed as numbers (proportions).

### Changes in LVMI, cardiothoracic ratio, and pre-dialysis BP between the DDD groups (DDD≤ 1, 1 < DDD ≤ 3, and DDD > 3)

LVMI, cardiothoracic ratio, pre-dialysis BP, and post-dialysis weight in the three groups at baseline and after 1 year, as well as the changes over 1 year, are presented in Table 4. Compared to patients with a lower DDD, those with a higher DDD showed a significant reduction in LVMI (*p* < .001) and cardiothoracic ratio (*p* = .033) over a period of 1 year. However, there was no significant change in pre-dialysis BP (*p* = .284) and post-dialysis weight (*p* = .058).

**Table 4.** LVMI, cardiothoracic ratio, pre-dialysis BP, and post-dialysis weight in the three groups at baseline and after 1 year, as well as the changes over 1 year.

Variables	DD*D* ≤ 1 (*n* = 20)	1 < DD*D* ≤ 3 (*n* = 159)	DD*D* > 3 (*n* = 61)	*p*–value
LVMI (g/m^2^)				
At baseline	75 (67–88)	116 (99–135)*	146 (119–172)*	<.001
At 1 year	92 (82–114)	118 (100–135)	128 (98–149)*	<.001
Change over a period of 1 year	20 (7–30)	–2 (–18–17)*	–26 (–45––8)*	<.001
Cardiothoracic ratio (%)				
At baseline	45 (42–48)	48 (46–53)*	51 (48–55)*	<.001
At 1 year	44 (42–49)	48 (45–51)	48 (46–51)*	.005
Change over a period of 1 year	.2 (–2.3–2.6)	–1.6 (–4.3–.6)	–2.7 (–6.0––.2)*	.033
Pre-dialysis blood pressure (mmHg)				
At baseline	158 (145–171)	159 (142–169)	153 (140–169)	.659
At 1 year	154 (139–162)	155 (143–170)	159 (141–168)	.180
Change over a period of 1 year	–7 (–27–11)	–1 (–19–16)	–3 (–20–22)	.284
Post–dialysis weight (kg)				
At baseline	65.1 (52.3–75.2)	56.0 (48.7–63.1)	56.0 (48.4–62.8)	.086
At 1 year	65.6 (53.3–76.2)	56.0 (49.0–62.7)	55.0 (49.2–63.0)	.064
Change over a period of 1 year	1.0 (–0.9–1.4)	0.4 (–0.5–0.8)	–0.4 (–1.4–0.9)	.058

LVMI: left ventricular mass index. The values are presented as medians (interquartile ranges).

*Indicates a significant difference compared with DDD ≤ 1, at a significance level of *p* < .05 (Bonferroni corrected Mann–Whitney post-hoc test).

### Changes in LVMI, post-dialysis weight, cardiothoracic ratio, and pre-dialysis BP between groups of DDD change (decrease, no change, and increase in DDD)

**Figure 2.**
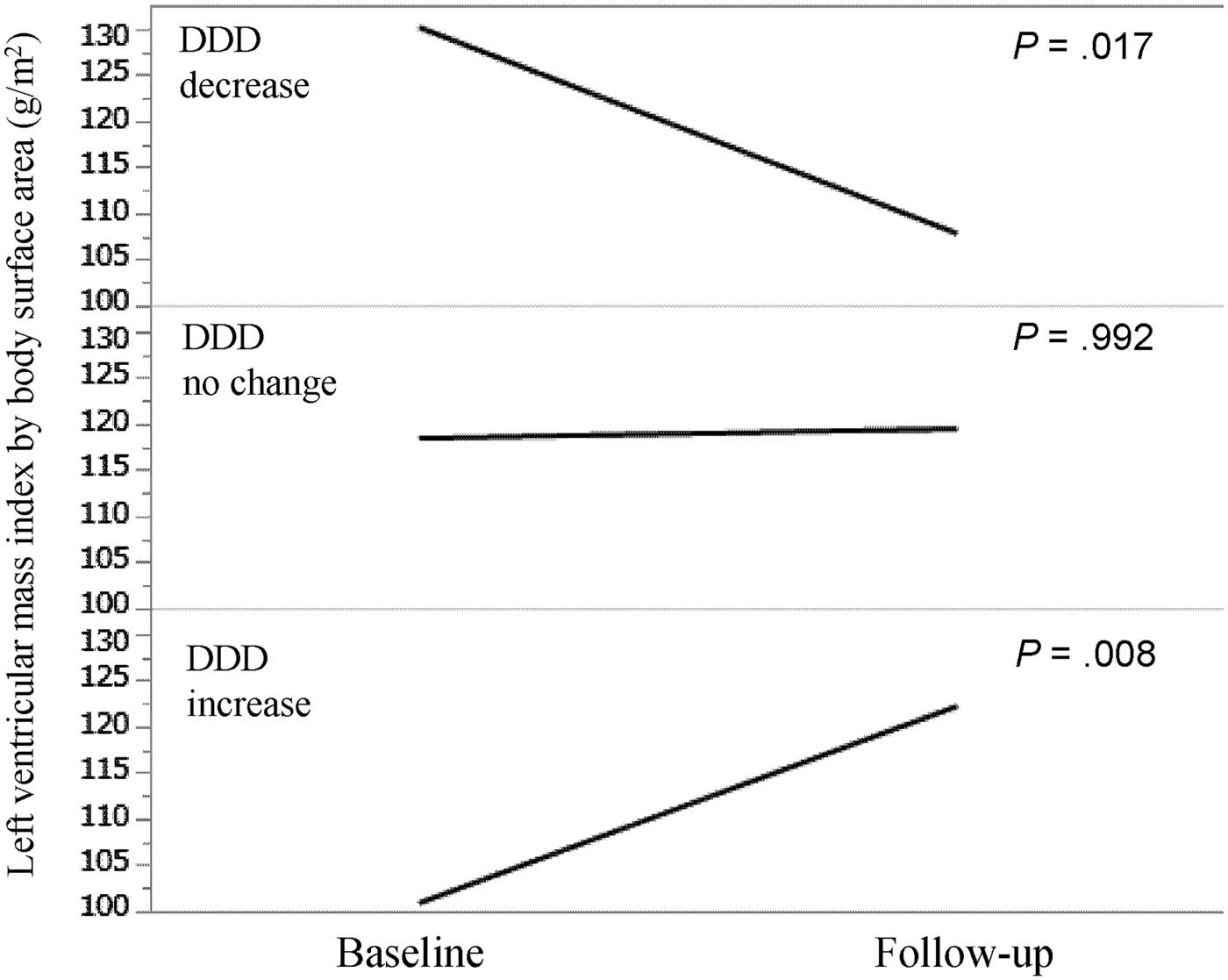
Left ventricular mass index by body surface area at baseline and follow-up stratified according to DDD changes. DDD: defined daily dose.

We compared the changes in the LVMI stratified according to the DDD changes (increase, no change, and decrease in DDD over 1 year) (Figure 2). In patients with decreased DDD, LVMI was significantly decreased from 130 g/m^2^ (interquartile range [IQR], 111–158 g/m^2^) at baseline to 108 g/m^2^ (IQR, 89–131 g/m^2^) at 1 year (*p* = .017). In patients exhibiting no change in DDD, no significant change in LVMI was observed (LVMI was 118 g/m^2^ [IQR, 99–143 g/m^2^] at baseline and 119 g/m^2^ [IQR, 99–143 g/m^2^] after 1 year) (*p* = .992). In patients with increased DDD, LVMI was significantly increased from 101 g/m^2^ (IQR, 85–117 g/m^2^) at baseline to 122 g/m^2^ (IQR, 110–138 g/m^2^) at 1 year (*p* = .008).

A significant decrease in the post-dialysis weight over the period of 1 year was observed in patients with decreased DDD compared to that in patients with increased DDD (*p* = .002) (Supplementary Table 2). Furthermore, a decrease in the cardiothoracic ratio over the period of 1 year was more frequently observed in patients with decreased than in those with increased DDD (*p* = .045) (Supplementary Table 2). No significant differences were observed in pre-dialysis BP, at baseline, 1 year, and over 1 year (*p* = .659, .180, and .252, respectively) (Supplementary Table 2).

### Clinical factors affecting LVMI changes

Univariate analysis revealed that higher LVMI and DDD at baseline were associated with a decrease in LVMI (*p* < .001). A decrease and an increase in DDD over a period of 1 year were significantly associated with reduced (*p* < .001) and increased LVMI (*p* < .001), respectively (Table 5). Furthermore, a change in the cardiothoracic ratio was significantly associated with a change in LVMI (*p* < .001).

**Table 5.** Univariate model for LVMI changes.

Candidate variables	β	SE	T	*p*-value
Age	–.093	.136	–.68	.496
Male sex (vs. female)	–.008	1.865	0	.997
Diabetes mellitus (vs. non-diabetes mellitus)	.744	1.772	.42	.675
BMI (kg/m^2^)	–.049	.496	–.1	.922
Pre-dialysis systolic BP (mmHg)	–.083	.077	–1.08	.282
Change in systolic BP (from baseline to 1 year) (mmHg)	.161	.053	3.03	.003
Hemoglobin (g/dL)	2.705	2.396	1.13	.260
Serum calcium (mg/dL)	–4.560	3.051	–1.49	.136
Serum phosphate (mg/dL)	.933	1.698	.55	.583
Serum intact PTH (pg/mL)	–.001	.019	–.07	.945
LDL-cholesterol (mg/dL)	–.024	.074	–.32	.748
Cardiothoracic ratio (%)	–.598	.330	–1.81	.071
Change in the cardiothoracic ratio over a period of 1 year (%)	1.475	.385	3.830	<.001
Changes in post-dialysis weight over a period of 1 year (kg)	2.486	1.562	1.59	.113
AVF (vs. AVG)	–1.848	2.844	–.65	.517
LVMI (g/m^2^) at baseline	–.392	.044	–8.9	<.001
LVEF (%) at baseline	.260	.160	1.62	.106
DDD (per day) at baseline	–16.642	1.659	–10.03	<.001
DDD change over a period of 1 year				
Decrease (vs. no change)	–24.160	3.171	7.62	<.001
Increase (vs. no change)	22.581	4.131	5.47	<.001

AVF: arteriovenous fistula; AVG: arteriovenous graft; BMI: body mass index; CRP: C-reactive protein; DDD: defined daily dose; LVEF: left ventricular ejection fraction; LVMI: left ventricular mass index; PTH: parathyroid hormone; RAS: renin-angiotensin system; SE: standard error of β; β: regression coefficient. The covariates ‘decrease’, ‘no change’, and ‘increase’ in DDD were included in the model. The covariate ‘No change in DDD’ was used as the reference value.

Multiple linear regression analysis was performed using all covariates that were assessed in the univariate model. It revealed that higher LVMI and cardiothoracic ratio values at baseline were associated with a decrease in LVMI (*p* < .001). An increase and a decrease in DDD over a period of 1 year were significantly associated with LVMI changes (*p* < .001), in addition to changes in systolic BP (*p* < .001) and cardiothoracic ratio (*p* < .001) (Table 6).

**Table 6.** Multiple model for changes in LVMI.

Candidate variables	β	SE	T	*p*-value
Age	–.042	.114	–0.370	.712
Male sex (vs. female)	–2.968	1.476	–2.010	.046
Diabetes mellitus (vs. non-diabetes mellitus)	1.375	1.476	.970	.334
BMI (kg/m^2^)	–.190	.440	–430	.666
Pre-dialysis systolic BP (mmHg)	–.260	.093	2.810	.006
Change in systolic BP (from baseline to 1 year) (mmHg)	.266	.0654	4.080	<.001
Hemoglobin (g/dL)	.446	1.828	.240	.808
Serum calcium (mg/dL)	–1.184	2.398	–.490	.622
Serum phosphate (mg/dL)	–.429	1.316	.330	.745
Serum intact PTH (pg/mL)	.019	.014	1.350	.177
LDL-cholesterol (mg/dL)	.063	.055	1.140	.256
Cardiothoracic ratio (%)	1.004	.343	2.930	.004
Change in the cardiothoracic ratio over a period of 1 year (%)	1.245	.373	3.330	.001
Changes in post-dialysis weight over a period of 1 year (kg)	–1.551	1.465	–1.060	.291
AVF (vs. AVG)	–.668	2.209	–.300	.763
LVMI (g/m^2^) at baseline	–.231	.058	–4.020	<.001
LVEF (%) at baseline	–.107	0.130	–.830	.410
DDD (per day) at baseline	–4.933	2.432	–2.030	.044
DDD change over a period of 1 year				
Decrease (vs. no change)	–17.386	2.424	–7.170	<.001
Increase (vs. no change)	16.192	2.738	5.910	<.001

AVF: arteriovenous fistula; AVG: arteriovenous graft; BMI: body mass index; CRP: C-reactive protein; DDD: defined daily dose; LVEF: left ventricular ejection fraction; LVMI: left ventricular mass index; PTH: parathyroid hormone; RAS: renin-angiotensin system; SE: standard error of β; β: regression coefficient. The covariates ‘decrease’, ‘no change’, and ‘increase’ in DDD were included in the model. The covariate ‘No change in DDD’ was used as the reference value.

## Discussion

This retrospective longitudinal cohort study showed that a change in the dose of antihypertensive drugs was significantly associated with changes in LVMI over a period of 1 year after HD initiation. Decreased antihypertensive drug use was significantly associated with reduced LVMI. Conversely, an increase in the use of antihypertensive drugs was significantly associated with LVMI progression. These results indicated that patients with decreased use of antihypertensive drugs became euvolemic, which led to reduced LVMI. Conversely, those who increased the use of antihypertensive drugs proceeded to volume retention, leading to LVMI progression. To the best of our knowledge, this was the first study evaluating the relationship between changes in the dose of antihypertensive drugs and changes in LVMI.

Previous observational cohort studies evaluated the predictors of LVH in dialysis patients. Higher pre-dialysis systolic BP [23], pre-dialysis mean arterial pressure [24], and intradialytic systolic BP [25] were associated with LVH. However, these studies did not evaluate the relationship between the prescription pattern of antihypertensive drugs (time-course changes in the dose of hypertensive drugs) and LVH progression.

Based on the method of control of BP in dialysis patients, a retrospective study reported that patients in the hypervolemic state had poor BP control, suggesting that antihypertensive drugs are ineffective in the hypervolemic state [12]. In contrast, the strict volume control group exhibited good BP control [13]. These results indicated that volume control is an important strategy for BP control in dialysis patients.

In the present study, patients with an increased use of antihypertensive drugs might have experienced hidden volume retention even when no obvious findings of volume overload were observed, leading to a greater effect on LVH progression than that in patients with a decreased dose of antihypertensive drugs. With respect to the volume status influence on LVMI, a cross-sectional study [14] reported that LVMI was lower in patients who demonstrated extracellular fluid volume reduction by dietary salt restriction and persistent ultrafiltration than in those who used an antihypertensive medication strategy despite the lack of a significant difference in BP between the groups. These findings suggested that the hypervolemic state may alter LVMI progression. However, due to the cross-sectional study design, causal relationships between the volume control and LVH reduction could not be determined. A randomized controlled study reported that the strict volume control group exhibited a reduction in BP and LVMI regression compared with the control group [26]. However, this study was based entirely on middle-aged patients (age, 51.6 ± 12.3 years), and the proportion of prescribed antihypertensive drugs was lower (23%) than that observed in a multicenter observational study (88%) [27]. It remains to be confirmed whether these results are confirmed in HD patients who are elderly or present a higher usage of antihypertensive drugs.

Although it is unclear whether persistent extracellular fluid retention without obvious findings of volume overload can still cause LVH progression when the BP is controlled through the use of antihypertensive drugs, we considered that fluid retention may be the most important contributing factor to LVMI progression in HD patients. Despite the importance of achieving an appropriate dry weight for BP control, the lack of a specific index for determining the appropriate volume status makes it challenging.

Our study revealed that a reduction in the use of antihypertensive drug use over a period of 1 year after HD initiation was correlated with a decrease in the cardiothoracic ratio on chest radiographs and a decreased dry weight. Furthermore, a decrease in the cardiothoracic ratio on chest radiographs was associated with a decrease in LVMI over a period of 1 year after HD initiation, suggesting that the euvolemic state of an appropriate dry weight could be approximated in patients who showed a reduction in the cardiothoracic ratio on chest radiographs and decreased dry weight. However, we did not use other tools, including bioimpedance plethysmography [26,28], relative plasma volume monitoring [29,30], measurement of the inferior cava diameter [12,31,32], and concentrations of plasma natriuretic peptides [22], to measure the volume status. In our practice, these methods were not regularly used. Future studies should employ these methods to evaluate the precise volume status. Furthermore, volume overload does not always equate to hypertension, because several factors, such as arterial stiffness, high renin states, the osmotic effects of glucose, and the nonosmotic effects of sodium could lead to a complex interplay [33]. It should be evaluated in future studies by including information concerning these factors.

DDD is useful when patients use the same medications before and after an intervention, with changes only in the dose. However, it may not be appropriate when there are changes in other anti-hypertensive drugs of different potencies. Generally, patients included in this study received the same medications during the study period. Especially, in cases where we aimed to change the potency of antihypertensive drugs, we first changed the dose, and not administered other anti-hypertensive agents. Regardless of the approach, when we could not attain a lower blood pressure, we provided other types of antihypertensive drugs.

Only five (2.1%) patients received medications of different potencies during the study period. Especially, in three out of these five patients, the medication changed from cilnidipine (10 mg) to nifedipine (20 mg), from cilnidipine (10 mg) to nifedipine (40 mg), and from cilnidipine (10 mg) to amlodipine (10 mg). However, all three patients received other RAS inhibitors. In the other two patients, the medication changed from losartan (25 mg) to valsartan (40 mg) and from losartan (25 mg) to olmesartan (20 mg). However, these two patients also received other calcium channel blockers. All five patients who were prescribed drugs of different antihypertensive potencies were included in the DDD increase group. Therefore, we considered that the aforementioned antihypertensive treatment changes might not have significantly influenced our results.

Our study had several limitations that are worth noting. First, although our work was, to a large extent, the most current study to examine the longitudinal changes in LVMI in HD patients, the sample size was small, and the observational period was short. Second, this study did not evaluate the difference in patient outcomes because of the short observation period. Larger longitudinal cohort studies should be performed to evaluate this issue. Third, because of the retrospective nature of the study, causal relationships between the reduction in the use of antihypertensive drugs and LVMI regression could not be determined. Fourth, LVMI was not assessed using cardiac magnetic resonance imaging (MRI), which can show the cardiac structures in detail [22,23,34]; therefore, our results should be validated by other studies using cardiac MRI. Fifth, those who exhibited LVMI regression over a period of 1 year demonstrated volume control and a possibility of improving uremic cardiomyopathy and anemia. However, our study did not precisely evaluate this possibility because of the study design employed.

Allowing for these methodological issues, our results suggested that in patients who had good BP control by receiving multiple antihypertensive drugs, reducing antihypertensive drug use as much as possible could be an important strategy to obtain strict volume control to attenuate LVH.

Our findings suggested that volume reduction, which leads to a decrease in the use of antihypertensive drugs, could be a therapeutic strategy for HD patients to prevent LVMI progression.

## Supplementary Material

Supplemental Material

Supplemental Material

## Data Availability

The authors confirm that the data supporting the findings of this study are available within this article and its supplementary materials (Supplementary Table 1).
