# Supplementary material for: Relationship between doses of antihypertensive drugs and left ventricular mass index changes in hemodialysis patients in a Japanese cohort
Source: Ren Fail. 2021 Jan 17;43(1):188–97. doi: 10.1080/0886022X.2021.1872626 (PMC7833083; doi:10.1080/0886022X.2021.1872626)
Supplement: Supplemental Material [file IRNF_A_1872626_SM5868.docx]

**Supplementary Table 2.** Post-dialysis weight, cardiothoracic ratio, and pre-dialysis systolic blood pressure at baseline and after 1 year, and the changes over the period of 1 year

| **Variables** | | **DDD decrease**  **(n=83)** | **DDD no change**  **(n=119)** | **DDD increase**  **(n=38)** | ***P* value** |
| --- | --- | --- | --- | --- | --- |
| Post-dialysis weight (kg) |  |  |  |  |  |
| At baseline | 57.2 (48.7–65.0) | 56.0 (48.8–65.0) | 56.0 (49.3–63.2) | .756 |  |
| At 1 year | 56.4 (48.8–66.1) | 55.1 (49.0–63.0) | 55.8 (49.8–63.6) | .808 |  |
| Change over a period of 1 year | -0.8 (-1.4–1.0) | 0.3 (-0.5–0.7)* | 0.8 (-0.3–-1.1)* | .002 |  |
| Cardiothoracic ratio (%) | |  |  |  |  |
| At baseline | | 49 (47–54) | 48 (46–54) | 49 (46–53) | .328 |
| At 1 year | | 48 (44–50) | 48 (45–51) | 48 (46–51) | .670 |
| Change over a period of 1 year | |  |  |  | .045 |
| Decrease (%) | | 62 (74.7) | 75 (63.0) | 20 (52.6) |  |
| Increase (%) | | 21 (25.3) | 44 (37.0) | 18 (47.4) |  |
| Pre-dialysis blood pressure (mmHg) | |  |  |  |  |
| At baseline | | 158 (145–171) | 159 (142–169) | 153 (140–169) | .659 |
| At 1 year | | 154 (139–162) | 155 (143–170) | 159 (141–168) | .180 |
| Change over a period of 1 year | | -8 (-24–16) | -5 (-27–22) | -2 (-26–31) | .252 |

Medians (interquartile ranges), categorical values are expressed as numbers (proportions).

* Indicates a significant difference compared with DDD decrease, at a significance level of P<.05 (Bonferroni corrected Mann–Whitney post hoc test).
